# Genome-Scale, Constraint-Based Modeling of Nitrogen Oxide Fluxes during Coculture of *Nitrosomonas europaea* and *Nitrobacter winogradskyi*

**DOI:** 10.1128/mSystems.00170-17

**Published:** 2018-03-13

**Authors:** Brett L. Mellbye, Andrew T. Giguere, Ganti S. Murthy, Peter J. Bottomley, Luis A. Sayavedra-Soto, Frank W. R. Chaplen

**Affiliations:** a Department of Botany and Plant Pathology, Oregon State University, Corvallis, Oregon, USA; b Department of Crop and Soil Science, Oregon State University, Corvallis, Oregon, USA; c Biological and Ecological Engineering, Oregon State University, Corvallis, Oregon, USA; d Department of Microbiology, Oregon State University, Corvallis, Oregon, USA; University of Michigan—Ann Arbor

**Keywords:** *Nitrobacter winogradskyi*, *Nitrosomonas europaea*, genome-scale, hydroxylamine, metabolic modeling, nitric oxide, nitrification, nitrous oxide

## Abstract

Nitrification, the aerobic oxidation of ammonia to nitrate via nitrite, emits nitrogen (N) oxide gases (NO, NO_2_, and N_2_O), which are potentially hazardous compounds that contribute to global warming. To better understand the dynamics of nitrification-derived N oxide production, we conducted culturing experiments and used an integrative genome-scale, constraint-based approach to model N oxide gas sources and sinks during complete nitrification in an aerobic coculture of two model nitrifying bacteria, the ammonia-oxidizing bacterium *Nitrosomonas europaea* and the nitrite-oxidizing bacterium *Nitrobacter winogradskyi*. The model includes biotic genome-scale metabolic models (iFC578 and iFC579) for each nitrifier and abiotic N oxide reactions. Modeling suggested both biotic and abiotic reactions are important sources and sinks of N oxides, particularly under microaerobic conditions predicted to occur in coculture. In particular, integrative modeling suggested that previous models might have underestimated gross NO production during nitrification due to not taking into account its rapid oxidation in both aqueous and gas phases. The integrative model may be found at https://github.com/chaplenf/microBiome-v2.1.

**IMPORTANCE** Modern agriculture is sustained by application of inorganic nitrogen (N) fertilizer in the form of ammonium (NH_4_^+^). Up to 60% of NH_4_^+^-based fertilizer can be lost through leaching of nitrifier-derived nitrate (NO_3_^−^), and through the emission of N oxide gases (i.e., nitric oxide [NO], N dioxide [NO_2_], and nitrous oxide [N_2_O] gases), the latter being a potent greenhouse gas. Our approach to modeling of nitrification suggests that both biotic and abiotic mechanisms function as important sources and sinks of N oxides during microaerobic conditions and that previous models might have underestimated gross NO production during nitrification.

## Abstract (precis)

Modern agriculture is sustained by application of inorganic nitrogen (N) fertilizer in the form of ammonium (NH_4_^+^). Up to 60% of NH_4_^+^-based fertilizer can be lost through leaching of nitrifier-derived nitrate (NO_3_^−^), and through the emission of N oxide gases (i.e., nitric oxide [NO], N dioxide [NO_2_], and nitrous oxide [N_2_O] gases), the latter being a potent greenhouse gas. Our approach to modeling of nitrification suggests that both biotic and abiotic mechanisms function as important sources and sinks of N oxides during microaerobic conditions and that previous models might have underestimated gross NO production during nitrification.

## INTRODUCTION

Modern industrialized agriculture is sustained by applications of inorganic nitrogen (N) fertilizer primarily in the form of ammonium (NH_4_^+^) (1). In this practice, up to 60% of NH_4_^+^-based fertilizer can be lost through microbial transformation and leaching of nitrate (NO_3_^−^) (2) and through the emission of N oxide gases (i.e., nitric oxide [NO], N dioxide [NO_2_], and nitrous oxide [N_2_O] gases) (3, 4). N_2_O is a potent greenhouse gas with a 298-fold-stronger atmospheric warming effect than CO_2_ and contributes to the depletion of the ozone layer (5). Nitrification is the key process controlling the initial transformation of NH_4_^+^-N in the environment and the efficiency of inorganic N uptake by plants (6).

Nitrification is generally carried out by chemolithotrophic microorganisms in a two-step process where ammonia (NH_3_) is oxidized to nitrite (NO_2_^−^) by ammonia-oxidizing bacteria (AOB) and ammonia-oxidizing archaea (AOA), and NO_2_^−^ is subsequently oxidized to NO_3_^−^ by nitrite-oxidizing bacteria (NOB) (6–10). In addition, the complete oxidation of NH_3_ to NO_3_^−^, comammox, was recently identified in bacteria previously characterized as NOB of the genus *Nitrospira* (11, 12). Nitrifying microorganisms have been shown to carry out denitrification under aerobic and microaerobic conditions producing NO and N_2_O (4, 13). Some studies have reported production of N_2_ gas by pure cultures of AOB, but a functional nitrous oxide reductase in AOB has not been demonstrated (13).

Representatives of the AOB, AOA, and NOB have the capacity to produce NO and N_2_O, but the exact mechanisms and overall contributions by each group of organisms are not well characterized (4, 13–17). The magnitude of nitrifier-derived emissions of N oxides generated by nitrification from soils and engineered environments are extremely variable and depend on a variety of environmental conditions such as the rate of nitrification, pH, temperature, and oxygen (O_2_), among other factors (4, 13, 18–21). Recent modeling efforts by Perez-Garcia et al. and others have sought to understand conditions that generate N oxides through single and multispecies metabolic network models of wastewater treatment systems (17, 22–24). Modeling N oxide production by simplified communities of model nitrifiers in both single culture and coculture, including abiotic reactions, can expand on previous work to better explain the mechanisms and conditions that affect N oxide gas emissions. Indeed, a recent report called for more controlled experiments on model microbial communities to inform modeling efforts (25). Our contribution to these efforts is the use of an integrative, genome-scale, constraint-based approach that considers both abiotic and biotic reactions to model complete nitrification by two model nitrifying bacteria, *Nitrosomonas europaea* and *Nitrobacter winogradskyi*, representing the AOB and NOB, respectively (26, 27).

Integrative genome-scale modeling provides a useful platform to investigate the biochemical pathways that function as sources and sinks of N oxide gas production during nitrification. Genome-scale, constraint-based modeling approaches apply physicochemical, spatiotemporal, and environmental constraints to a reaction network that captures the materials and energy processing activities of a microorganism (28, 29). These models assume that the condition of homeostasis or steady-state operation reached by a microorganism is the result of an optimized physiological response for a given set of environmental conditions (30, 31). Whereas most microbial growth conditions in soils and other systems are dynamic, constraint-based models require a pseudo-steady-state assumption for analysis. To account for this, dynamic conditions can be approximated using integrative modeling with dynamic flux balance analysis (dFBA), which places the steady-state constraint-based formulism inside a discrete time step dynamic approximation that uses Michaelis-Menten kinetics to simulate nutrient uptake (30–33).

In this study, the physiological responses of *N. europaea* and *N. winogradskyi* during experimental batch culturing were modeled under aerobic and microaerobic conditions in closed systems using an integrative genome-scale, constraint-based approach. To improve the model simulations, we developed an integrated model of both biotic reactions from the genome-scale model and abiotic reactions, particularly gas phase and aqueous oxidation of NO in the presence of O_2_. Experimental data were modeled to identify potential biotic and abiotic sources and sinks of N oxide gases during complete nitrification. Modeling of complete nitrification in this system suggests that AOB, NOB, and abiotic mechanisms function as important sources and sinks of N oxides during microaerobic conditions due to competition for dissolved O_2_. The results of our integrated modeling approach suggest that previous models might have underestimated gross NO production during nitrification.

## RESULTS

### Nitrification in a closed system produces NO_x_ and N_2_O.

**FIG 1:**
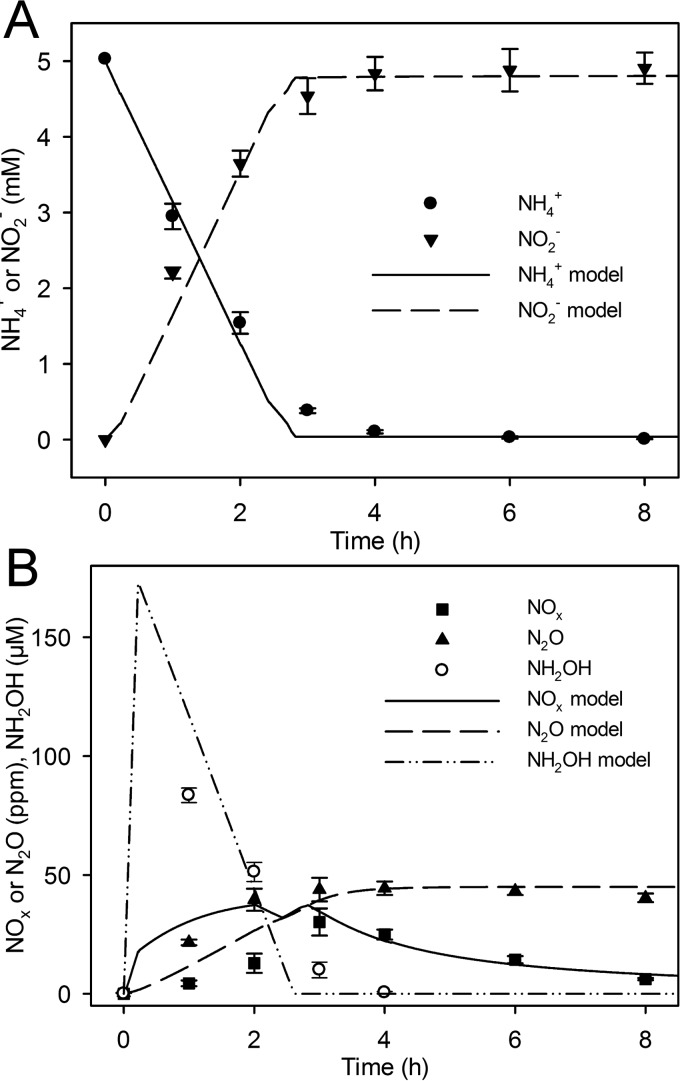
N fluxes during *N. europaea* culture. Data points (symbols) represent experimental data, and lines show model simulations. (A) Liquid NH_4_^+^ concentration (in millimolar) and liquid NO_2_^−^ concentration (in millimolar) (*y* axis) are shown over time (in hours) (*x* axis). (B) Headspace NO_x_ concentration (in parts per million [ppm]) (*y* axis), headspace N_2_O concentration (ppm) (*y* axis), and aqueous NH_2_OH (in micromolar) (*y* axis) over time (in hours) (*x* axis). Symbols indicate experimental values. Experimental values are means ± standard deviations of the means (error bars) (*n* = 4).

**FIG 2:**
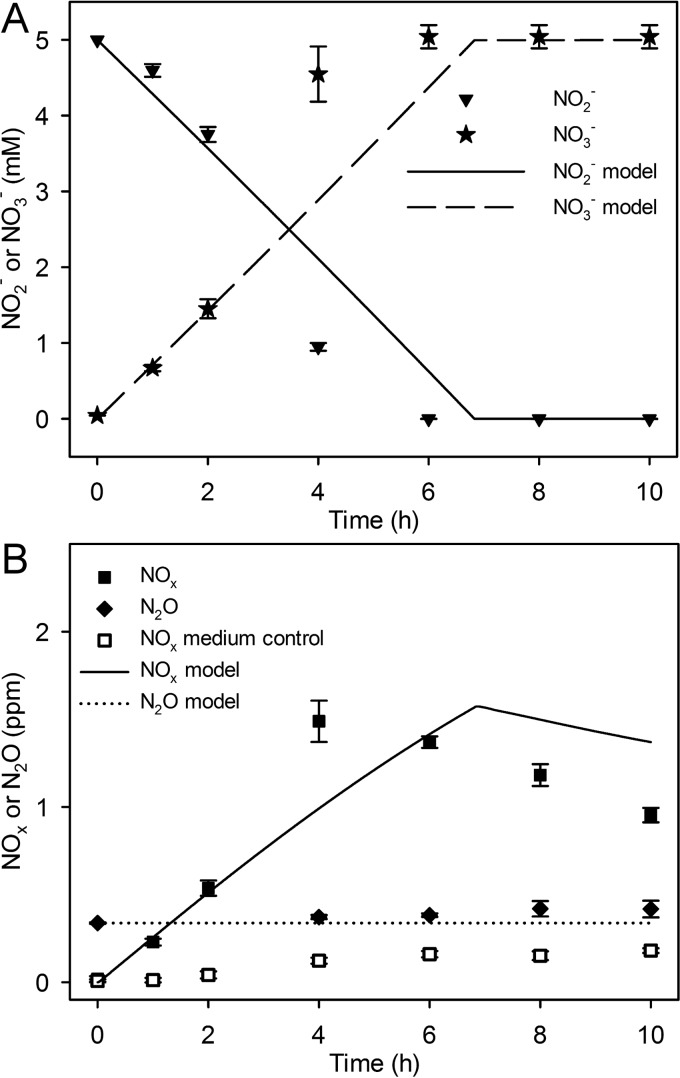
N fluxes during *N. winogradskyi* culture. Data points (symbols) represent experimental data, and lines show model simulation. (A) Liquid NO_2_^−^ concentration (in millimolar) and liquid NO_3_^−^ concentration (in millimolar) (*y* axis) measured over time (in hours) (*x* axis). (B) Headspace NO_x_ concentration (ppm), headspace N_2_O concentration (ppm), and headspace NO_x_ in abiotic medium controls over time (h) (*x* axis). Symbols indicate experimental values. Experimental values are the means ± standard deviations of the means (error bars) (*n* = 4).

**FIG 3:**
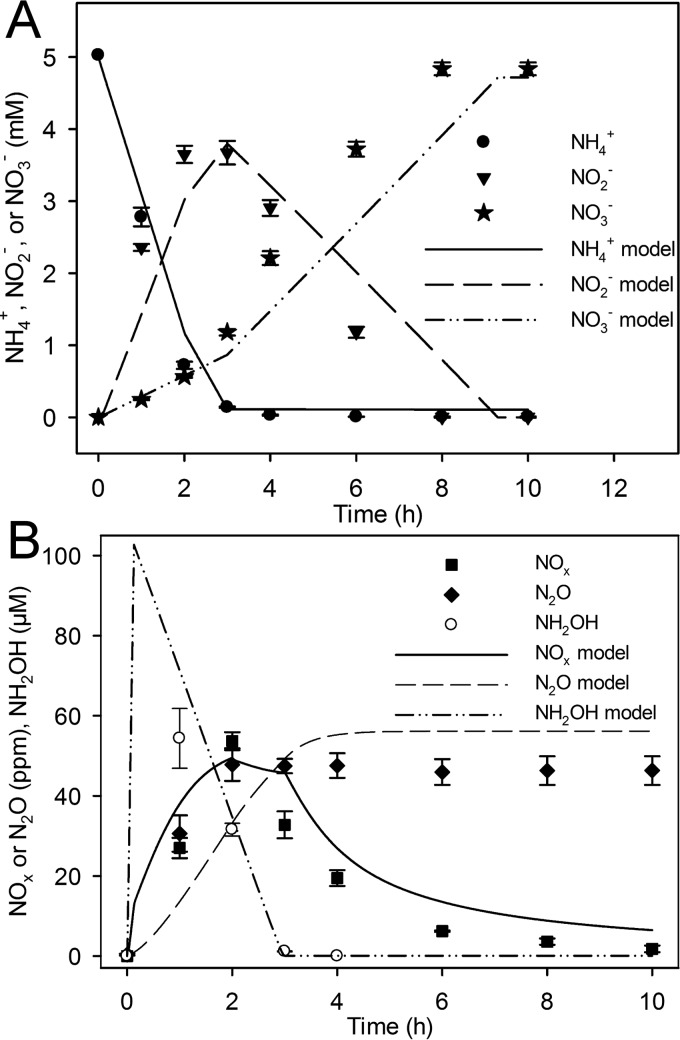
N fluxes during coculture of *N. europaea* and *N. winogradskyi*. Data points represent experimental data, and lines show model simulations. (A) Liquid NH_4_^+^ concentration (mM), liquid NO_2_^−^ concentration, and liquid NO_3_^−^ concentration (mM) (*y* axis) measured over time (h) (*x* axis). (B) Headspace NO_x_ concentration (ppm) (*y* axis), headspace N_2_O concentration (ppm) (*y* axis), and aqueous NH_2_OH (µM) (*y* axis) over time (h) (*x* axis). Symbols indicate experimental values. Experimental values are the means ± standard deviations of the means (error bars) (*n* = 4).

Both single cultures of *N. europaea* and *N. winogradskyi* and the coculture of both nitrifiers produce NO and NO_2_ (collectively NO_x_) and N_2_O during aerobic nitrification (Fig. 1B, 2B, and  3B). *N. europaea* produced more net NO_x_ and N_2_O in single culture than *N. winogradskyi* did, but the coculture produced more N oxide gases (NO_x_ and N_2_O) than the sum of the N oxide gases in the single cultures (Fig. 1 to  3). In single culture, *N. europaea* actively produced both NO_x_ and N_2_O up to 30.2 ± 5.7 and 42.1 ± 0.9 ppm, respectively, during active NH_3_ oxidation (Fig. 1B). However, net production of both N_2_O and NO_x_ stopped when NH_4_^+^ was exhausted and was followed by NO_x_ concentrations decreasing over time (Fig. 1A and B). Ammonia oxidation in single cultures of *N. europaea* acidified the medium from approximately pH 7.80 to 6.95 (data not shown).

In contrast, *N. winogradskyi* produced statistically significant amounts of N oxide (1.5 ± 0.12 ppm of NO_x_ and 0.08 ± 0.04 ppm of N_2_O; *P* < 0.05 for N_2_O produced in the first 4 h) that were minute by comparison to *N. europaea* (Fig. 2B and 1B). Net accumulation of NO_x_ occurred when NO_2_^−^ oxidation was initiated, but net consumption of NO_x _commenced only after 4 h when 80% of the NO_2_^−^ had already been consumed (Fig. 2A and B). There was no significant change in the pH of single cultures of *N. winogradskyi* (data not shown).

The coculture of *N. europaea* and *N. winogradskyi* produced approximately 53.7 ± 2.2 and 47.8 ± 4.1 ppm of NO_x_ and N_2_O, and the sum of the net N oxide gases was greater than for the single cultures combined (*P* < 0.005) (Fig. 3B). Maximum accumulation of N oxide gases peaked after 2 h before NH_3_ oxidation was completed and when NO_2_^−^ oxidation was initiated (Fig. 3A and B). Net consumption of NO_x_ in the coculture occurred more rapidly than from single cultures (Fig. 1B, 2B, and 3B). Interestingly, the coculture appeared to consume the NH_4_^+^ more rapidly than the *N. europaea* culture, and yet it took 8 h to consume the accumulated NO_2_^−^ (Fig. 1A and 3A). The pH of the cocultures acidified from approximately 7.80 to 7.08 (data not shown).

### Model simulations predict N oxide production and hydroxylamine accumulation during aerobic nitrification.

**FIG 4:**
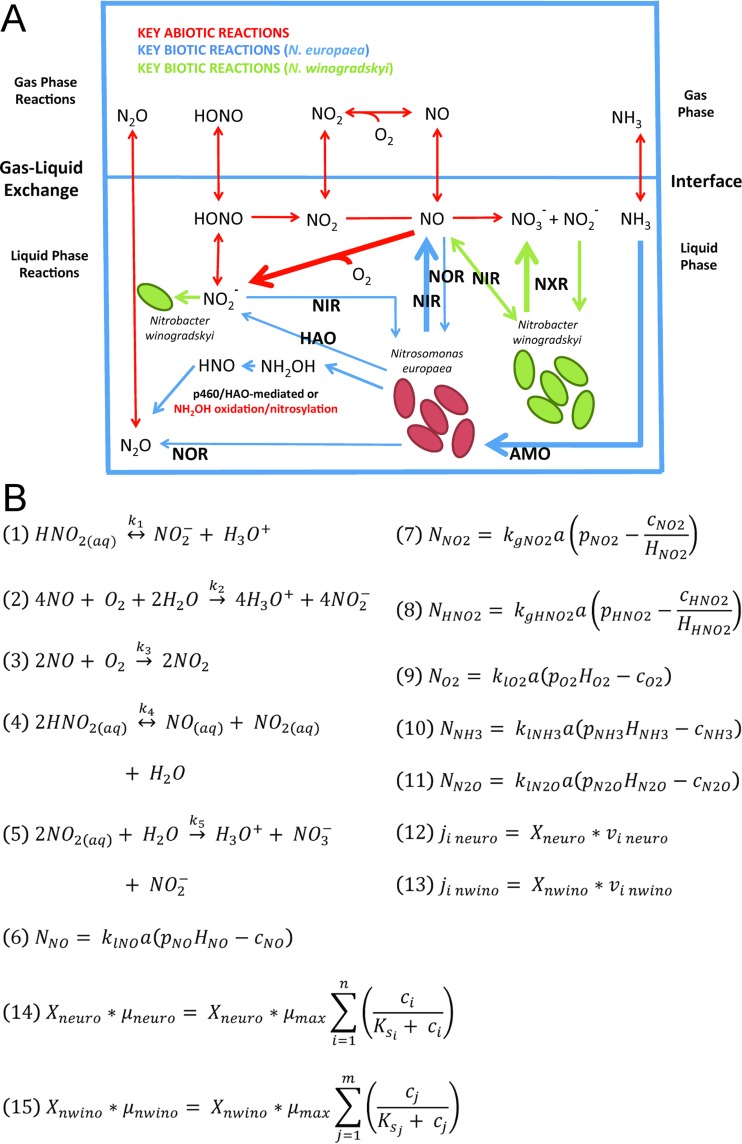
Conceptual model of N distribution in coculture. (A) The schematic represents key biotic and abiotic reactions modeled during a coculture of *N. europaea* and *N. winogradskyi*. Key abiotic reactions (red) and key biotic reactions carried out by *N. europaea* (blue) and *N. winogradskyi* (green) are shown. The thickness of the lines represents the relative significance of the reaction. Biotic enzymatic reactions are labeled as follows: AMO, ammonia monooxygenase; HAO, hydroxylamine dehydrogenase; NXR, nitrite oxidoreductase; NIR, nitrite reductase; NOR, nitric oxide reductase; p460, cytochrome P460. (B) Equations describing key schematic biotic and abiotic reactions. Abbreviations: aq, aqueous; *N*_*x*_, gas exchange of component *x* across gas-liquid interface (M s^−1^); *a*, interfacial area (m^2^); *k*_lx_, liquid side gas mass transfer coefficient of *x* (m^−2^ s^−1^); *k*_gx_, gas side gas mass transfer coefficient of *x* (m^−2^ s^−1^); *p*_*x*_, partial pressure of *x* (atm); *H*_*x*_, Henry’s law constant of *x* (M atm^−1^); *c*_*x*_, liquid phase concentration of *x* (M); *µ*_max_, maximum growth rate under nonnutrient limitation (s^−1^); *μ*, growth rate under nutrient limitation (s^−1^); *K*_sx_, substrate concentration of *x* at half-maximum growth rate (M); *X*, cell mass (gDCW liter^−1^); *j*_*i*_, molar flux of chemical species *i* from organism (mmol liter^−1^ h^−1^); neuro, *N. europaea*; nwino, *N. winogradskyi*. Note that growth and species production rates for *N. europaea* and *N. winogradskyi* were calculated with genome-scale models through linear programming with maximization of growth rates at each time step.

The integrative model was calibrated for each single culture and coculture case to track the experimental data (Fig. 1 to 3). Specifically, constraints were placed on nitrite reductase (NIR) and nitric oxide reductase (NOR) activity in the *N. europaea* genome-scale model and on NIR activity in the *N. winogradskyi* genome-scale model. In addition, uptake rates for NH_4_^+^ and NO_2_^−^ in the model were adjusted to match the corresponding experimental measurements shown in Fig. 1 to 3. After genome-scale model calibration, coculture simulations were used to determine sources and sinks of N oxides during nitrification (abiotic and biotic reactions are listed in Fig. 4B). Two different candidate simulations for *N. europaea* were considered: candidate model 1 calibrated the model to maximize biomass production, and candidate model 2 calibrated the model to maximize NO_2_^−^ production for the first 2 h of the experiment and to maximize biomass for the remaining time (see Fig. S1 in the supplemental material). Both model simulations suggested cycling between enzymatic and abiotic sources and sinks of N oxides particularly during active NH_3_ oxidation. However, candidate simulation model 1 predicted transient accumulation of hydroxylamine (NH_2_OH) in the growth medium, while candidate simulation model 2 did not (Fig. S1).

10.1128/mSystems.00170-17.2 FIG S1  Candidate model predictions of hydroxylamine (NH_2_OH) during coculture of *N. europaea* and *N. winogradskyi*. The lines indicate modeling of predicted aqueous NH_2_OH (in micromolar) (*y* axis) over time (in hours) (*x* axis). Candidate model 1 maximizes biomass production, and candidate model 2 maximizes NO_2_^−^ production for 2 h followed by biomass production. Download FIG S1, TIF file, 0.1 MB. Copyright © 2018 Mellbye et al. 2018 Mellbye et al. This content is distributed under the terms of the Creative Commons Attribution 4.0 International license.

A second set of experiments was carried out to refine the model, and these experiments validated the accumulation of NH_2_OH in the batch culture system (Fig. 1B and 3B). Extracellular NH_2_OH accumulated to approximately 83.5 ± 3.1 µM and 54.4 ± 7.5 µM after 1-h incubation of *N. europaea* single culture and coculture, respectively (Fig. 1B and 3B). After 1 h, net consumption of NH_2_OH occurred and was followed by complete consumption at 4 h. The final model simulations presented in Fig. 1 and 3 predict all N oxide fluxes by prioritizing maximum NH_3_ uptake during the initiation of the experiment for 15 min before changing to maximizing both biomass and NH_3_ uptake rate. These changes were based on previous work documenting *N. europaea*’s ability to quickly take up and oxidize NH_3_ during recovery from starvation, such as the N- and energy-limited steady-state chemostat cells used in this study (34, 35).

**FIG 5:**
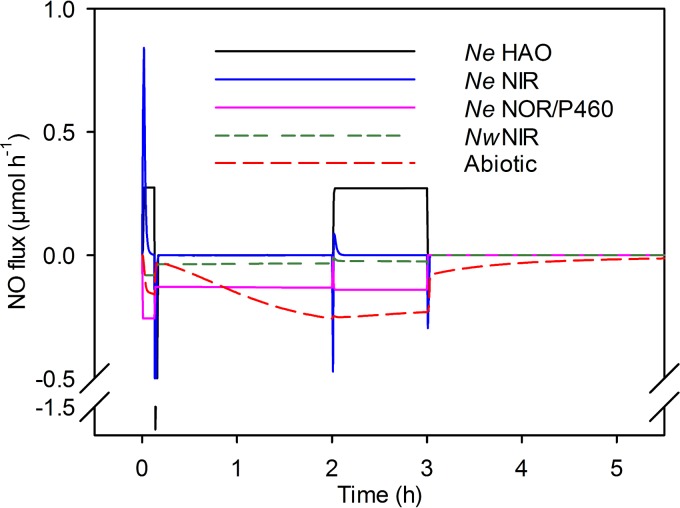
NO sources and sinks during coculture nitrification by *N. europaea* and *N. winogradskyi*. Instantaneous NO production or consumption (in micromoles hour^−1^) (*y*  axis) by *N. europaea* HAO, *N. europaea* NIR, *N. europaea* NOR/cytochrome P460, *N. winogradskyi* NIR, and abiotic reactions modeled over time (h) (*x*  axis). *Ne*, *N. europaea*; *Nw*, *N. winogradskyi*.

Modeling predicted that NIR and hydroxylamine dehydrogenase (HAO) activities were the sources of NO production in *N. europaea* and that production began as O_2_ concentrations decreased after inoculation and initiation of NH_3_ oxidation for both coculture models (Fig. 3 and Fig. S2). Most of the NO was produced by *N. europaea* via NIR and HAO (Fig. 5). As shown in Fig. 2B, abiotic production of NO_x_ was insignificant, and the integrative model did not predict a significant contribution to total N oxide flux by gaseous nitrous acid (HONO). NO and N_2_O production by *N. europaea* were concurrent in most cases. Model predictions indicated that the enzymatic source of N_2_O is dependent on the O_2_ status of the culture (Fig. S2). Early in the incubation, the model predicted that *N. europaea* NOR was the principal source of N_2_O with significant contributions from cytochrome P460 (both represented as NO consumption in Fig. 5). The model suggests that abiotic N oxide production from NH_2_OH was not a significant source (data not shown).

10.1128/mSystems.00170-17.3 FIG S2  Model prediction of dissolved oxygen during culturing of *N. europaea* and *N. winogradskyi*. Lines indicate predicted dissolved oxygen gas (O_2_ [in millimolar]) over time (in hours) based on genome-scale, integrative modeling of nitrification. *N. europaea* (*Ne*) model, *N. winogradskyi* (*Nw*) model, and *N. europaea* and *N. winogradskyi* coculture model are shown. Download FIG S2, TIF file, 0.2 MB. Copyright © 2018 Mellbye et al. 2018 Mellbye et al. This content is distributed under the terms of the Creative Commons Attribution 4.0 International license.

### NO_x_ accumulation is a complex function of both biotic and abiotic reactions and of dissolved O_2_ depletion in liquid culture.

To explain the accumulation of NO_x_ in the headspace of the liquid cultures, an integrative model encompassing biotic and abiotic reactions of N oxide species was necessary. The dynamic reaction network, including biotic and abiotic reactions, is summarized in Fig. 4. In particular, reaction 2 (Fig. 4B), aqueous abiotic oxidation of NO to NO_2_^−^, is essential to explain NO_x_ accumulation during nitrification. Model simulations suggest that gross production of NO is much higher than net NO_x_ accumulation measured in the headspace, since both aqueous phase oxidation of NO to NO_2_^−^ and enzymatic reactions consume NO (Fig. 5).

Both experimental data and model simulations suggested that *N. europaea* is the predominant producer of both NO and N_2_O. To overcome abiotic oxidation of NO to NO_2_^−^ in aqueous solution and to account for the peak in NO_x_ observed during single culture, *N. winogradskyi* would need to produce NO at a maximum rate of approximately 130 nmol h^−1^. On the other hand, according to model simulations, maximum NO production in the *N. europaea* peaks at approximately 1 µmol h^−1^. While the *N. winogradskyi* culture produces relatively minute amounts of N_2_O, the genome-scale model does not include biotic N_2_O production by *N. winogradskyi* due to the lack of a known gene encoding NOR (27). The majority of NO_x_ production observed in *N. europaea* pure culture was dependent on NIR activity with consumption being through abiotic oxidation of NO to NO_2_^−^ and biotic consumption via NIR, NOR, and HAO (Fig. 5). We interpret the higher levels of NO_x _in the coculture being due to both *N. europaea* and *N. winogradskyi* producing NO under potentially O_2_ diffusion-limited conditions caused by the cell densities (Fig. 2B and Fig. S2). NO_x_ consumption from the headspace can be explained through both biotic and abiotic reactions in coculture (Fig. 5).

## DISCUSSION

### Integrative modeling with a reaction network containing both abiotic and biotic reactions provides new insights into nitrification coupling.

To date, most genome-scale and metabolic models of nitrification have focused on biotic reactions (17, 22, 23, 36). While this approach simplifies the construction of models, it neglects the importance of abiotic chemistry in the N cycle (13). By integrating a model of biotic reactions informed by genome-scale models of *N. europaea* and *N. winogradskyi* with an abiotic model of N species reaction kinetics, both production and consumption of N oxide gases during nitrification could be explained more accurately. In order for NO_x_ to be detected in the gas phase, our integrative modeling approach predicted that net production of NO would have to be greater than the abiotic oxidation of NO to NO_2_^−^ in the aqueous phase of the culture. In addition, N oxide production is exacerbated under O_2_ transport-limited conditions, but the rate constants for NO oxidation are high enough that significant inhibition of the oxidation reaction was not observed at the lowest concentrations of O_2_ predicted.

### Both AOB and NOB contribute to NO_x_ and N_2_O production through different enzymatic pathways.

AOB, such as *N. europaea*, are thought to carry out NH_3_ oxidation by oxidizing NH_3_ to NH_2_OH by ammonia monooxygenase (AMO) and NH_2_OH to NO_2_^−^ by HAO with a gross yield of four electrons. Recent studies have suggested that the sole product of HAO oxidation of NH_2_OH is NO with a yield of three electrons, further suggesting that oxidation to NO_2_^−^ is either abiotic or carried out by an unknown enzymatic step (37, 38). While this study suggests that abiotic oxidation of NO is a significant NO sink, a true yield of four electrons resulting from NO oxidation to NO_2_^−^ by an unknown NO oxidase would result in increased biomass production by *N. europaea*. The genome-scale model presented in this work suggests that NO oxidation to NO_2_^−^ by NIR, as previously demonstrated (39), is unlikely, as it negatively affects the ATP balance, and further experiments are needed to identify a potential candidate final enzymatic step.

While production of N oxides by *N. europaea* has been well studied both through experimentation and modeling (4, 18, 22, 37, 38), production of N oxides by *N. winogradskyi* is more cryptic. Early studies reported both production of NO and N_2_O and consumption of NO by strains of *N. winogradskyi* and *Nitrobacter vulgaris* (40, 41), but only NO consumption, not production, was shown in one later study on *N. winogradskyi* (42). Poughon et al. suggested that production of NO_2_^−^ from NO by NIR in the cytoplasm of *N. winogradskyi* was thermodynamically feasible under high NO_2_^−^ conditions (43). Production of NO from NO_2_^−^ in the periplasm is followed by diffusion of NO into the cytoplasm where NO is converted back to NO_2_^−^, followed by a final conversion of NO_2_^−^ to NO_3_^−^ by nitrite oxidoreductase (NXR) (43). This results in a net translocation of protons from the cytoplasm to the periplasm and has a positive effect on ATP production (43). The integrated model for *N. winogradskyi* required a significant change in the energy model to reflect NO_2_^−^ oxidation rates during the batch culture experiments. At 3 h, a simpler model in which NIR activity was present only in the periplasm was shifted to the more complex Poughon model, with NIR activity in both the cytoplasm and periplasm, to reflect experimental data suggesting low initial rates of NO_2_^−^ oxidation (43). The advantage of the Poughon model to the organism is that the ATP yield increases from 0.667 mmol ATP per mmol NO_2_^− ^to 1.53 mmol ATP per mmol NO_2_^−^. However, maintenance energy also increases (from 8 to 18.5 mmol ATP gDCW^−1^ h^−1^ where gDCW stands for gram [dry cell weight]).

Our current model does not consider acyl-homoserine lactone quorum sensing (QS) regulation of NO production proposed in recent work by Mellbye et al. due to insufficient data on the kinetic effects of QS regulation (16). The genome-scale model also does not consider the minute N_2_O production observed by *N. winogradskyi*, since its genome does not contain any known NOR-encoding gene. Future studies are needed to determine the regulatory effects of QS on NO fluxes and whether QS effects need to be incorporated into a future energy model for *N. winogradskyi*.

Interestingly, during complete nitrification in the coculture, the sum of the N oxide gases produced by the coculture was greater than the sum produced by the single cultures despite having the same substrate and cell densities. Model simulations of dissolved O_2_ suggest that the O_2_ demand of NH_3_ oxidation temporarily creates a microaerobic environment that is exacerbated by the activity of the NOB (see Fig. S2 in the supplemental material). On the basis of published data of O_2_ affinity and coculture experiments, the O_2_ competition places higher stress on *N. winogradskyi* (44, 45). In addition, the models suggest that the switches in NO_x_ production rates observed for both organisms are a function of changes in O_2_ levels (Fig. S2).

Despite a lower dissolved O_2_, the NH_3_ oxidation rate in coculture is actually higher than the rate of the single culture, and the NO_2_^−^ oxidation rate is much lower than the rate in single culture until NH_3_ oxidation ceases (Fig. 1 to 3). The observation of increased NH_3_ uptake and oxidation rates could be due to increased NO production under microaerobic conditions by *N. europaea* as predicted by the integrative model. In addition, since less NH_2_OH accumulated in the coculture, the model suggests that a portion of the accumulated NH_2_OH may have been converted to N oxides by cytochrome P460. The observation of decreased NO_2_^−^ oxidation rates could be due to increased NO consumption by *N. winogradskyi*; further experiments are needed to test these hypotheses. Genome-scale modeling of these data provided further insight into the nitrification process by indicating which pathways or abiotic reactions cause accumulation of NO_x_ and N_2_O.

Our experimental data and model simulations add further support to reports that increased NH_3_ oxidation rate causes increased N_2_O production. NH_3_ oxidation and growth rate data generated in previous studies suggests that an increased NH_3_ oxidation rate leads to uncoupling of NH_3_ oxidation from growth, forcing *N. europaea* to direct electrons to NIR and NOR to regenerate reductant (21, 46–48). In addition, the integrative model reported here suggests different energy outcomes for the cell based on abiotic oxidation of NO to NO_2_^−^. These simulations suggest that *N. europaea* loses substantial energy during microaerobic NH_3_ oxidation, as NO is produced by NIR and either abiotically oxidized to NO_2_^−^, recaptured by HAO, or reduced to N_2_O by NOR.

### Application of biotic and abiotic models to complex systems.

The integrative model developed here is a first step toward modeling of N oxide emissions from more complex environmental systems, such as soils. Abiotic reactions can have a profound effect in environmental systems, and modeling efforts in these systems will need to take these reactions into account. For example, both abiotic and biotic factors are important for NO and N_2_O production in drying soils, particularly in the face of a potentially warming climate (5, 49). Another important factor influencing N_2_O production in environmental systems are gas diffusion constraints (50). Integration of these abiotic factors will lead to more accurate models of N oxide production from environmental systems.

Finally, genome-scale modeling of other nitrifiers are needed, since AOA can be the dominant NH_3_ oxidizer, and NO_2_^−^ oxidizers of the genus *Nitrospira* are often the most numerous NOB (9, 51). Among AOB, the genus *Nitrosospira* is usually most numerous in soils, and genomic data are available for assembly into genome-scale metabolic models (52–54). Perez-Garcia et al. (17) constructed a stoichiometric metabolic model of several AOB and NOB based on genomic data and published studies of nitrifying mixed cultures in wastewater treatment, but specific kinetic information and genome-scale models of many species are still lacking. Before genome-scale metabolic models of these microorganisms can be fully completed, growth and energy yield parameters and nutrient and O_2_ affinity data are needed. In addition, experimental corroboration of the energy models that inform the genome-scale models needs to occur. Recent work with *Nitrospira* enrichment cultures has begun to provide this important information for NOB (55). The integration of a genome-scale constraint-based model and abiotic reaction model presented in this work is a key step toward making meaningful predictions in complex systems.

## MATERIALS AND METHODS

### Bacterial strains and routine culture conditions.

*Nitrosomonas europaea* (ATCC 19718), *Nitrobacter winogradskyi* (Nb-255), and a coculture of *N. europaea* and *N. winogradskyi* were routinely cultivated at 30°C in batch and chemostat culture as previously described (56). Chemostat cultures were maintained in steady state at a dilution rate of 0.015 h^−1^. All cultures were routinely screened for heterotrophic contamination by plating 200-µl aliquots of culture on Luria-Bertani (LB) agar plates.

### Experimental batch culturing.

Experimental batch cultures were established by harvesting and washing cells from steady-state chemostat single cultures of *N. europaea* and *N. winogradskyi* and a steady-state coculture chemostat of *N. europaea* and *N. winogradskyi*. Harvested cells were suspended in 5 ml of experimental batch growth medium at the appropriate cell density in 160-ml serum vials. *N. europaea*, *N. winogradskyi*, and coculture experimental cultures were suspended to an optical density at 600 nm (OD_600_) of 0.2, 0.05, and 0.25, respectively. The cell densities were chosen to emulate coculture cell densities of *N. europaea* and *N. winogradskyi*. The relative cell densities of *N. europaea* and *N. winogradskyi* during coculture were previously determined (56). Batch experiments were assayed in a closed-batch culture system with sufficient O_2_ in the headspace and CO_2_ in the medium, supplied as Na_2_CO_3_, for complete N species oxidation and C fixation, respectively. Experimental cultures were capped with gray butyl stoppers, crimp sealed, and incubated for 10 h at 30°C with shaking at 200 rpm. Two hundred microliters of vial culture was routinely sampled to measure extracellular ammonium/ammonia (NH_4_^+^/NH_3_), hydroxylamine (NH_2_OH), NO_2_^−^, NO_3_^−^, OD_600_, and/or pH as outlined below. NO and NO_2_ (collectively NO_x_) and N_2_O concentrations were routinely measured in the headspace as outlined below.

Experimental batch mineral salts medium for *N. europaea* contained 2.5 mM (NH_4_)_2_SO_4_, 0.75 mM MgSO_4_, 0.1 mM CaCl_2_, 12.5 mM KH_2_PO_4_, 1.25 mM NaH_2_PO_4 _⋅_ _H_2_O, 2.3 mM Na_2_CO_3_, and the following trace elements: 10 µM FeCl_3_ chelated with EDTA (16.7 µM), 1 µM CuSO_4_, 0.6 µM Na_2_MoO_4 _⋅_ _2H_2_O, 1.59 µM MnCl_2 _⋅_ _4H_2_O, 0.6 µM CoCl_2 _⋅_ _6H_2_O, and 0.96 µM ZnSO_4 _⋅_ _7H_2_O. The medium for *N. winogradskyi* was the same formulation as the *N. europaea* medium except that it contained 5 mM NaNO_2_ instead of (NH_4_)_2_SO_4_. The pH of the experimental batch medium was adjusted to 7.8.

### Analytical methods.

NH_4_^+^/NH_3_ and NO_2_^−^ concentrations were measured by chemical assays as previously described (57). NO_3_^−^ concentration was determined by high-pressure liquid chromatography (HPLC) as previously described (56). Extracellular NH_2_OH concentration was measured by chemical assay as previously described (58, 59). NO and NO_2_ (NO_x_) concentrations in the headspace were measured using a portable NO_2_ analyzer/NO_x_ converter (LMA-3D and LNC-3D; Unisearch Associates Ltd., Concord, Ontario, Canada), and N_2_O concentration was measured by gas chromatography as previously described (16, 21).

### Model structure.

The base modeling framework was provided through dynamic multispecies metabolic modeling (DyMMM) (33) using dynamic flux balance analysis (dFBA), which provides a discretized dynamic modeling environment for metabolic models. Monod (Michaelis-Menten)-type models of substrate uptake and the effects of inhibitory compounds provided the interface between models of the environment (the medium in the bioreactor) and the microorganism, allowing the modeling of dynamic cell growth and function systems. The dynamic shell of the integrative models included differential equations for the biotic and abiotic reaction networks and mass transfer relationships between the gas and liquid phases within the batch bioreactors; these equations both informed and were informed by genome-scale, metabolic models for *N. europaea* and *N. winogradskyi*. A schematic of the experimental system and summary of the combined reaction network is shown in Fig. 4. The full reaction network is detailed in the supplemental material (Data Sets S1 to S4).

10.1128/mSystems.00170-17.5 DATA SET S1  Genome-scale model of *N. europaea*. This data set contains the complete set of curated reactions, model compounds, biomass calculations, cellular maintenance calculations, ion content determinations, lipid content determinations, and associated tables and references for the genome-scale model of *N. europaea*. Download DATA SET S1, XLS file, 0.9 MB. Copyright © 2018 Mellbye et al. 2018 Mellbye et al. This content is distributed under the terms of the Creative Commons Attribution 4.0 International license.

10.1128/mSystems.00170-17.6 DATA SET S2  Genome-scale model of *N. winogradskyi*. This data set contains the complete set of curated reactions, model compounds, biomass calculations, cellular maintenance calculations, ion content determinations, lipid content determinations, and associated tables and references for the genome-scale model of *N. winogradskyi*. Download DATA SET S2, XLS file, 0.8 MB. Copyright © 2018 Mellbye et al. 2018 Mellbye et al. This content is distributed under the terms of the Creative Commons Attribution 4.0 International license.

10.1128/mSystems.00170-17.7 DATA SET S3  Supplemental equations, rate constant data, and mass transfer coefficient calculations for the integrative model. This data set contains additional equations, rate constant data, and mass transfer coefficient calculations, and references for these data. Download DATA SET S3, XLSX file, 0.5 MB. Copyright © 2018 Mellbye et al. 2018 Mellbye et al. This content is distributed under the terms of the Creative Commons Attribution 4.0 International license.

10.1128/mSystems.00170-17.8 DATA SET S4  Energy models and maintenance energy. This data set contains equations and reactions incorporated in the energy models for *N. europaea* and *N. winogradskyi* and chemostat experiments used to calculate maintenance energies. Download DATA SET S4, XLSX file, 0.3 MB. Copyright © 2018 Mellbye et al. 2018 Mellbye et al. This content is distributed under the terms of the Creative Commons Attribution 4.0 International license.

### Metabolic network reconstruction.

The genome-scale, stoichiometric models (iFC578, *Nitrosomonas europaea*; iFC579, *Nitrobacter winogradskyi*) were based upon the Department of Energy (DOE) Joint Genome Institute (JGI) sequences for *N. europaea* and *N. winogradskyi* with automatic annotation and model building through the SEED, “a peer-to-peer environment for genome annotation” (60, 61), followed by hand annotation using the Kyoto Encyclopedia of Genes and Genomes (KEGG) database (http://www.genome.jp/kegg/) (62). The models were developed as previously described (63). Briefly, the models were calibrated either to maximize biomass production or energy substrate uptake, and constraints were applied to uptake rates of ammonia and nitrite based on previously published kinetic parameters and experimental data generated in this study (42, 64, 65). The *V*_max_ values for ammonia and nitrite uptake were calculated based on the slopes of the ammonia and nitrite consumption curves for each experimental time segment; the *K*_*m*_ values for uptake were published values (66) or selected such that the steady-state concentration value for these components matched the final concentrations measured experimentally for each component. *V*_max_ and *K*_*m*_ values are reported in Data Set S3. The biomass equation for both genome-scale reconstructions was derived from the *Escherichia coli* biomass equation reported for iAF 1260 (67) and modified based on lipid composition measurements of *N. europaea* and *N. winogradskyi* (Text S1 and Table S1). For the study of coupled growth dynamics, the model equations for microbial energetics from previous work were adapted to develop genome-scale models of *N. europaea* (iFC578) and *N. winogradskyi* (iFC579) (7, 8, 43). Schematics of the energetic pathways for *N. europaea* and *N. winogradskyi* under modeled conditions are shown in supplemental material (Data Sets S1 to S4). The genome-scale models required non-growth-associated maintenance (NGAM) energy requirements to be calculated based on energy model assumptions and chemostat experiments. The NGAM for *N. europaea* was 52.82 mmol ATP gDCW^−1^ h^−1 ^(where gDCW stands for gram [dry cell weight]) based on previous chemostat experiments (21) and using the energy model shown in the supplemental material (Data Set S4). The NGAM for *N. winogradskyi* was 8 mmol ATP gDCW^−1^ h^−1^ for 3 h, followed by 18.52 mmol ATP gDCW^−1^ h^−1^ for the rest of the experiment. A change in the energy model and associated maintenance energy was required 3 h into the culturing experiments to account for the changes in the experimental nitrite oxidation rate by *N. winogradskyi*. The advantage of the new model is that the ATP yield is higher; however, it is not possible to implement at lower NO_2_^−^ oxidation rates because of the increased rate of NGAM maintenance energy required by the organism. Maintenance energies were determined using data from chemostat experiments performed for the current study and previous work (Data Set S4) (8, 43). The growth-associated maintenance (GAM) energy for both organisms was determined based on the method of Balagurunathan et al. and found to be 1,060 mmol ATP gDCW^−1^ h^−1^ (68). The genome-scale models were formatted in Systems Biology Markup Language (SBML) level 3 version 1.0 (sbml.org) and was read into MatLab using SBMLToolbox (version 4.1) (69) and libSBML (version 5.6.0) (70). The model files in Excel format are available in the supplemental material, and SBML, GAMS, and MatLab files are available at GitHub (https://github.com/chaplenf/microBiome-v2.1) (Data Sets S1 to S4).

10.1128/mSystems.00170-17.1 TEXT S1  Supplemental materials and methods, model calibration and structure flowchart, and supplemental references. Supplemental materials and methods describe fatty acid analyses of *N. winogradskyi* and *N. europaea*. The model calibration and structure flowchart further describe calibration and structure of the integrative model. Download TEXT S1, PDF file, 0.2 MB. Copyright © 2018 Mellbye et al. 2018 Mellbye et al. This content is distributed under the terms of the Creative Commons Attribution 4.0 International license.

10.1128/mSystems.00170-17.4 TABLE S1  Fatty acid methyl ester (FAME) profile of *N. europaea*. Download TABLE S1, DOCX file, 0.02 MB. Copyright © 2018 Mellbye et al. 2018 Mellbye et al. This content is distributed under the terms of the Creative Commons Attribution 4.0 International license.

### Simulations.

MatLab version 2014b (MathWorks, Inc.) running the Cobra Toolbox v3.0 (https://arxiv.org/abs/1710.04038) was used to integrate the set of differential equations describing the distribution and reaction network of N-containing compounds that results from the dissolution of NaNO_2_ in water, including abiotic pH-dependent reactions, as shown in the supplemental material (Text S1 and Data Set S3). The model pH was fixed at 7.4 to reduce the number of differential equations and simplify the model, since the pH decreased from approximately 7.8 to 7.0 in the experiments. The equation set was stiff and used the ode15s function of MatLab. Parameter values are based on previous studies and are detailed in the supplemental material (Text S1 and Data Set S3). The genome-scale models were called by MatLab as needed during integration and were written in the General Algebraic Modeling System (GAMS) (https://www.gams.com/products/introduction/). The integrative model follows much the same file structure and conceptual model as DyMMM (33). In brief, the run file calls ode15s, which in turn calls a model file during each ode15s time step; the integrator and not the user selects the time steps except for system output. The first part of the model file called by ode15s calculates the uptake rates for the different substrates for the GAMS using Monod-type relationships. Next, the program calls the GAMS organism files in order to provide the model-predicted organism outputs for inclusion with the abiotic reactions. A flowchart detailing the model algorithm can be found in the supplemental material (Text S1). Finally, there are metabolite and nonmetabolite balance reaction calculations for the batch before the program exits the ode15s model file. The integrative models used mass transfer and Henry’s law coefficients were determined as described in the supplemental material (Data Set S3).
