# Supplementary figures and images for: Genome-Scale, Constraint-Based Modeling of Nitrogen Oxide Fluxes during Coculture of Nitrosomonas europaea and Nitrobacter winogradskyi
Source: mSystems. 2018 Mar 13;3(3):e00170-17. doi: 10.1128/mSystems.00170-17 (PMC5864417; doi:10.1128/mSystems.00170-17)

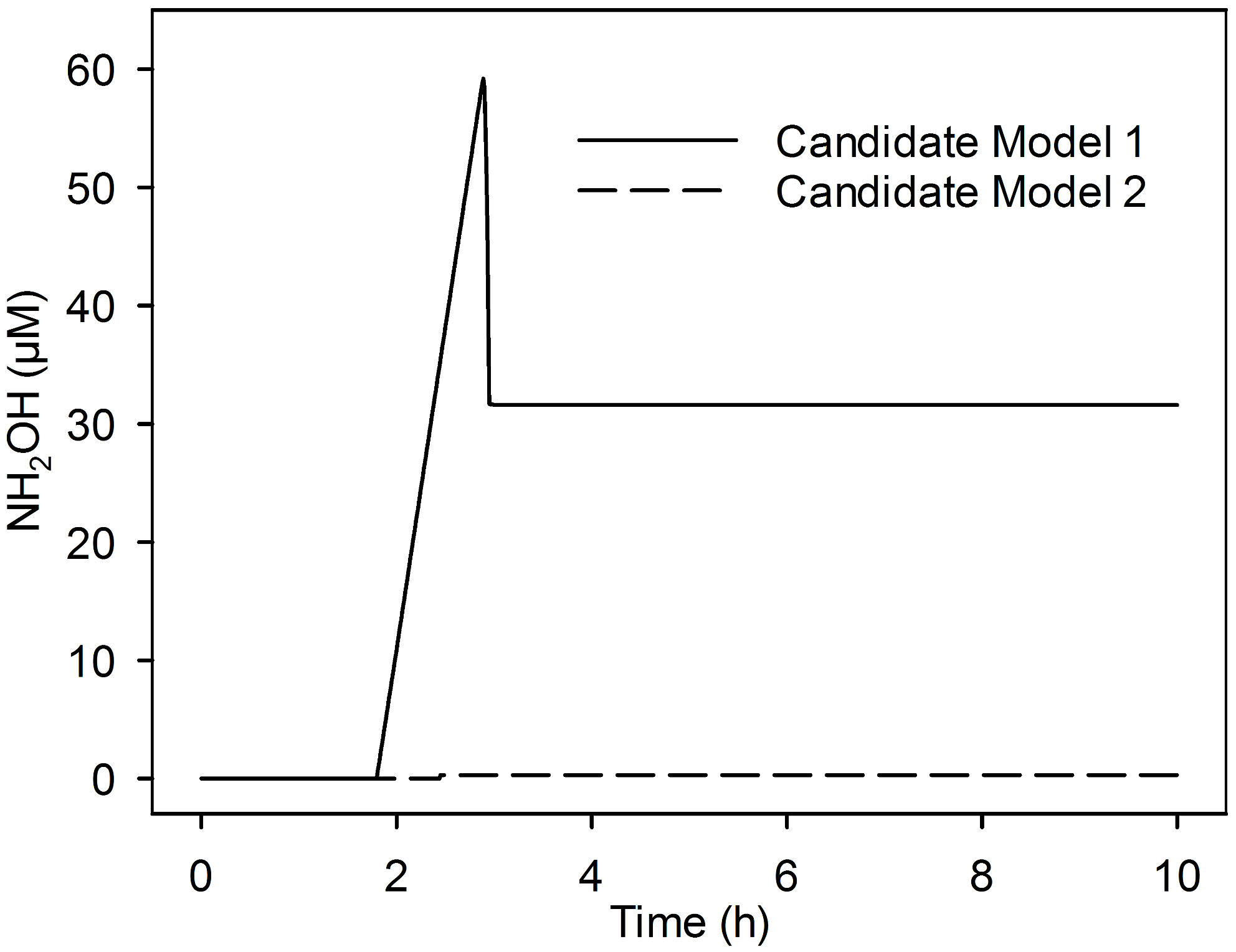

Supplement: FIG S1 [file sys003182205sf1.tif]

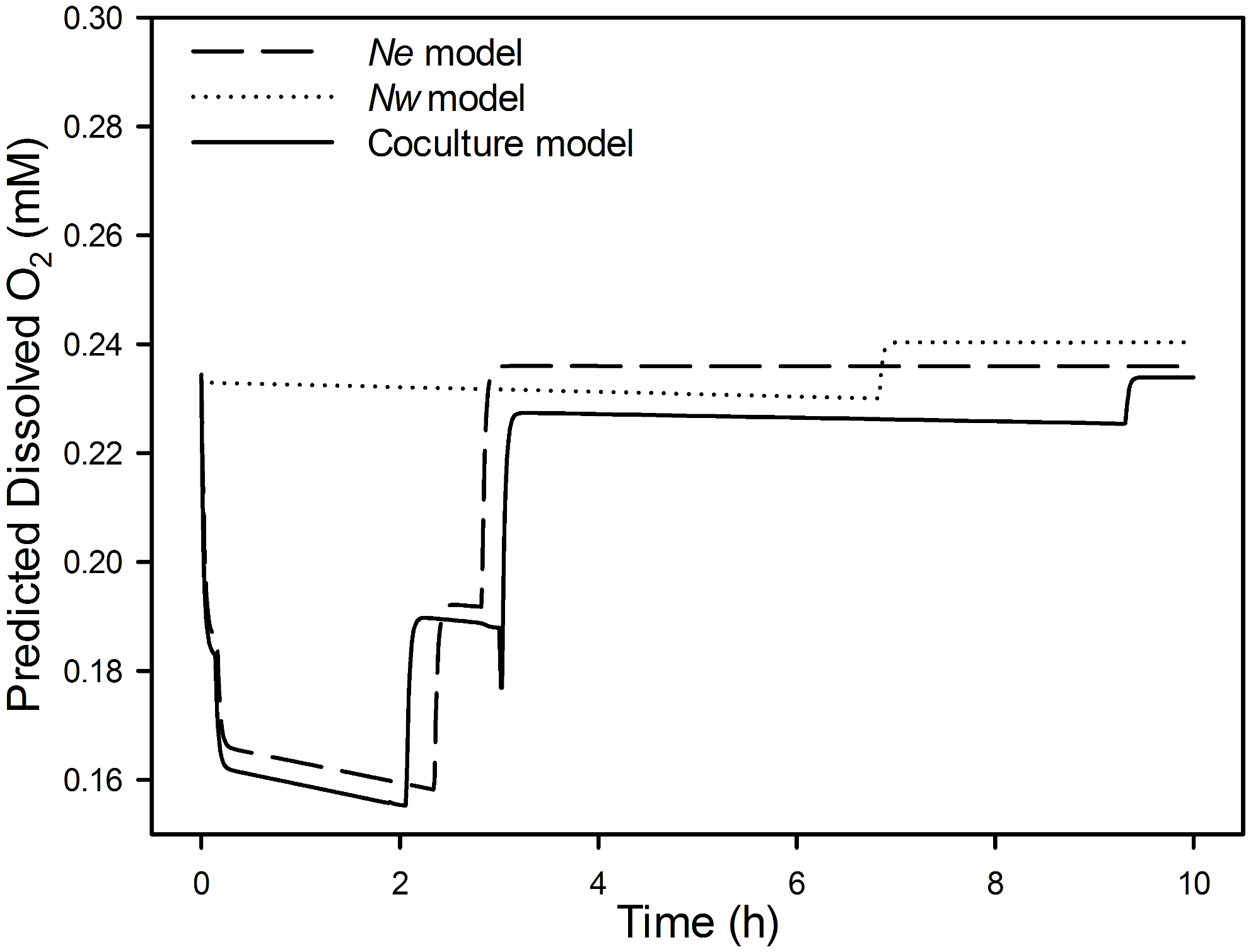

Supplement: FIG S2 [file sys003182205sf2.tif]
