# Supplementary material for: Genome-Scale, Constraint-Based Modeling of Nitrogen Oxide Fluxes during Coculture of Nitrosomonas europaea and Nitrobacter winogradskyi
Source: mSystems. 2018 Mar 13;3(3):e00170-17. doi: 10.1128/mSystems.00170-17 (PMC5864417; doi:10.1128/mSystems.00170-17)
Supplement: TEXT S1 [file sys003182205s1.pdf]

Supplementary information

Genome-scale, constraints-based modeling of nitrogen oxide fluxes during co-culture of *Nitrosomonas europaea* and *Nitrobacter winogradskyi*.

Brett L. Mellbye[a#], Andrew Giguere[b], Ganti S. Murthy[c], Peter J. Bottomley[b,d], Luis A. Sayavedra-Soto[a], and Frank Chaplen[c#].

[a]Department of Botany & Plant Pathology, Oregon State University, Corvallis, Oregon, USA[a]; [b]Department of Crop & Soil Science, Oregon State University, Corvallis, Oregon, USA[b]; [c]Biological & Ecological Engineering, Oregon State University, Corvallis, Oregon, USA[c]; [d]Department of Microbiology, Oregon State University, Corvallis, Oregon, USA[d].

[#]Address correspondence to Brett L. Mellbye, mellbyeb@oregonstate.edu, and Frank Chaplen, frank.chaplen@oregonstate.edu

This PDF file includes:

Supplementary Methods

Model Calibration and Structure Flow Chart

Supplementary References

SUPPLEMENTAL MATERIALS AND METHODS

Fatty acid analysis. FAME profiles for *Nitrobacter winogradskyi* were taken from previous work (1). Stationary-phase culture samples of *Nitrosomonas europaea* were submitted to Microbial ID, Inc. (MIS, Microbial ID, Inc. (MIDI), Newark, Delaware, USA) for fatty acid methyl ester (FAME) generation, extraction, and identification by gas chromatography as described for *N. winogradskyi* (1). FAME profiles were generated by Microbial ID, Inc. (MIS, Microbial ID, Inc. (MIDI), Newark, Delaware, USA) using Sherlock 6.1 as described (2).

MODEL CALIBRATION AND STRUCTURE

We calibrated the integrative model by varying the upper bound on the NIR reaction and switching on or off denitrification reactions catalyzed by HAO. We show the figures for the initial model calibration in Supplemental Materials (Figure S1), which predicted HAO production, but later in the batch culture than ultimately measured experimentally. The final model calibration disabled N_2 production by HAO, included a short time period (15 minutes) where ammonia uptake was maximized, and then changed to maximize biomass production.

The integrative model (microbiome v2.1) may be found at GitHub (github.com). Matlab Vers. 2014b (Mathworks, Inc.) running the Cobra Toolbox v3.0 (https://arxiv.org/abs/1710.04038) was used to integrate the set of differential equations found in HONOModelODE. Later versions of Matlab have a modified GAMS interface and will not run microBiome v2 as currently formatted. The structure of

microBiomev2.1 is shown in the flow chart that follows. Download the Cobra Toolbox v3.0 and the latest version of the GAMS IDE (www.gams.com). Rewrite the path for the various files called by Neuro_model; Neuro_model1; and Nwino_model to the model directory. Make sure that the GAMS and model directories are in the Matlab path and launch runFile.m."

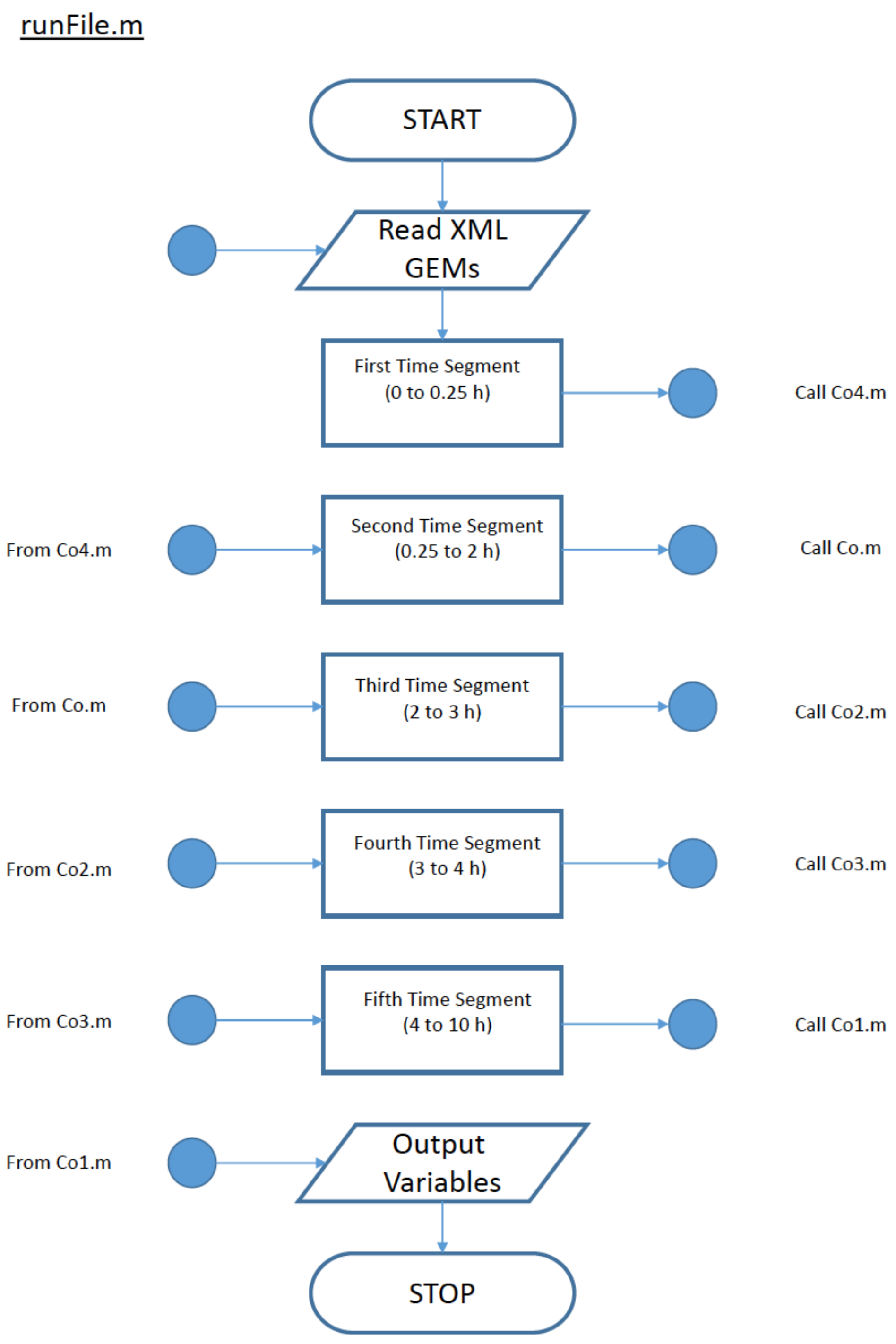

Co*.m

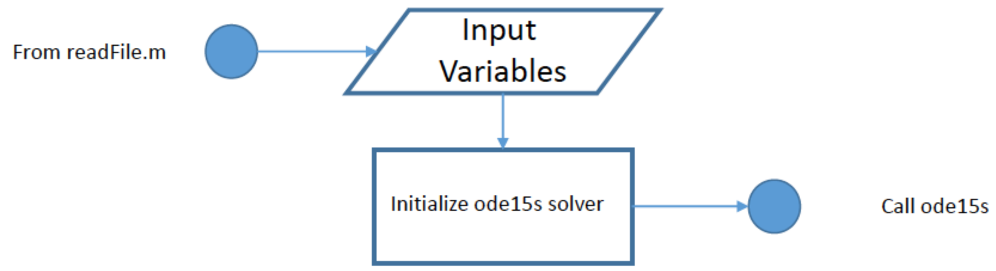

coCulture_ode*.m

This is the model file used by ode15s

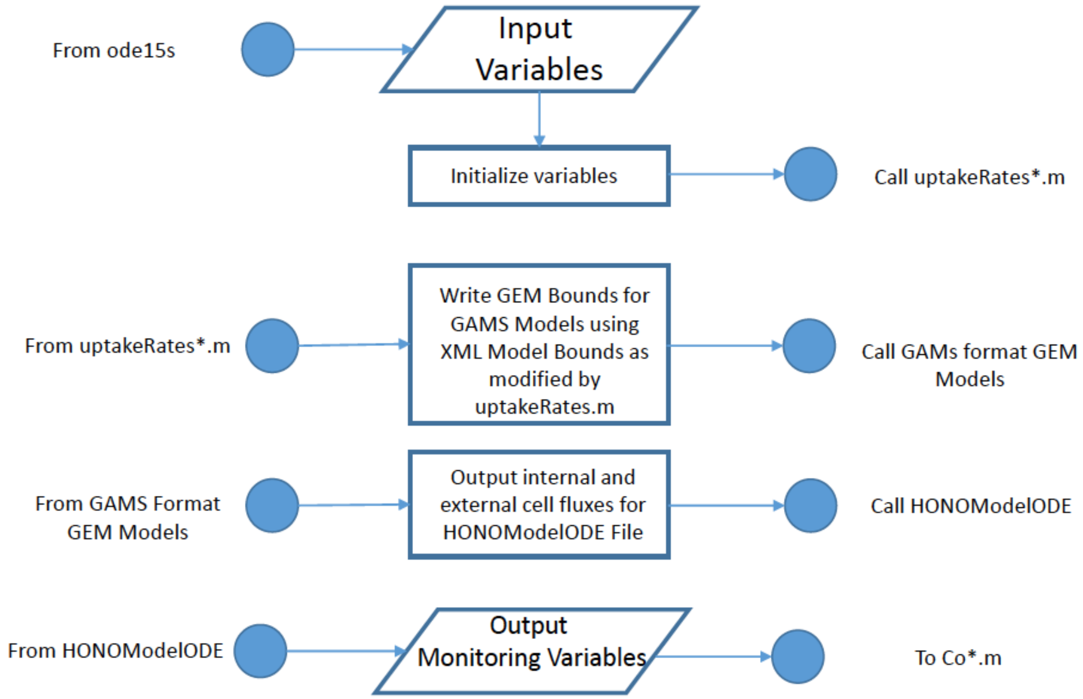

HONOModelODE.m

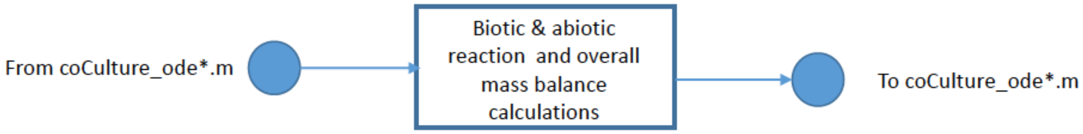

SUPPLEMENTAL REFERENCES

1. **Mellbye BL, Bottomley PJ, Sayavedra-Soto LA.** 2015. Nitrite-oxidizing bacterium *Nitrobacter winogradskyi* produces N-acyl-homoserine lactone autoinducers. Appl Environ Microbiol **81:**5917-5926.

2. **Assih EA, Ouattara AS, Thierry S, Cayol JL, Labat M, Macarie H.** 2002. *Stenotrophomonas acidaminiphila* sp. nov., a strictly aerobic bacterium isolated from an upflow anaerobic sludge blanket (UASB) reactor. Int J Syst Evol Microbiol **52:**559-568.
