# Supplementary material for: Genome-Scale, Constraint-Based Modeling of Nitrogen Oxide Fluxes during Coculture of Nitrosomonas europaea and Nitrobacter winogradskyi
Source: mSystems. 2018 Mar 13;3(3):e00170-17. doi: 10.1128/mSystems.00170-17 (PMC5864417; doi:10.1128/mSystems.00170-17)
Supplement: TABLE S1 [file sys003182205st1.docx]

**Table S1.** FAME profile of *Nitrosomonas europaea*.

| RT*^a^* | ECL*^b^* | Peak Name | Percent | Comment1*^c^* | Comment2*^d^* |
| --- | --- | --- | --- | --- | --- |
| 0.7250 | 6.6105 |  | ---- |  |  |
| 0.7353 | 6.6793 | SOLVENT | ---- |  |  |
| 2.1894 | 13.9100 | 14:1 w5c | 0.13 | -0.006 |  |
| 2.2157 | 13.9997 | 14:0 | 0.18 | 0.000 |  |
| 2.5992 | 15.2359 | 14:0 2OH | 0.51 | 0.003 |  |
| 2.7916 | 15.8422 | Sum In Feature 3 | 66.43 | 0.002 | 16:1 w7c/  16:1 w6c |
| 2.8419 | 16.0006 | 16:0 | 32.49 | 0.001 |  |
| 3.4745 | 17.9997 | 18:0 | 0.26 | 0.000 |  |
| ---- | ---- | Summed Feature 3 | 66.43 | 16:1 w7c/  16:1 w6c | 16:1 w6c/  16:1 w7c |

*^a^*Retention time. *^b^*Equivalent chain lengths values. *^c^*ECL deviation or unresolved peak(s) in summed features. *^d^*Best guess for unresolved peak(s).
